# Supplementary material for: Variations in face experiences during the COVID-19 pandemic affect infants’ preference for their mother’s face
Source: PLoS One. 2026 Feb 11;21(2):e0340620. doi: 10.1371/journal.pone.0340620 (PMC12893562; doi:10.1371/journal.pone.0340620)
Supplement: S3 File — (DOCX) [file pone.0340620.s003.docx]

**Alteration of face experiences during the COVID-19 pandemic affects infants’ preference for the mother’s face.**

Supplemental Materials

**Survey Questions in Experiment 3**

The survey included the following questions:

1. Composition of family members living together
2. [if you do not live together with the relatives] Where do most of the relatives live?

Options: Living in the same municipality/Living in the same prefecture/Living outside the prefecture/others

1. [if you do not live together with the relatives] How long does it take you to get to the relative’s house from your house by your usual means of transportation? (e.g., it takes about thirty minutes to get to the grandparents’ house by car.)
2. How often did the family members living together with infants wear face masks in the house when they interacted with the infants?

Options: Every time wore/Almost wore/Neither/Almost never wore/Never wear.

1. How many relatives visit the house?
2. How many times did the relatives visit the home? (e.g., the grandmother came to 5 times)
3. How often did visiting relatives wear face masks when interacting with the infants?

Options: Every time wore/Almost wore/Neither/Almost never wore/Never wore.

1. How many people other than the relatives visit the home?
2. How many times did people other than relatives visit the home? (e.g., a mother’s friend came to twice)
3. How often did people other than the relatives who visited the home wear face masks when interacting with the infants?

Options: Every time wore/Almost wore/Neither/Almost never wore/Never wore.

1. How many times have you visited relatives and/or friends?
2. How many people did the infants meet in total when visiting relatives and/or friends?
3. How often did relatives and/or friends wear face masks in their house when interacting with the infants?

Options: Every time wore/Almost wore/Neither/Almost never wore/Never wore.

Using the answers to the questions above, we calculated: (1) The number of family members living together, and (2) The number of people not wearing face masks to whom infants were exposed. If infants’ parents indicated that a person/people “never wore” or “almost never wore” face masks, they were considered as a person/people who did not wear a face mask. It included family members living together as well as relatives and friends who visited the participants’ house and relatives and friends whom the participants’ family visited. After comparing the mother-prefer group (infants showing mother’s face preference over 50% in the unmasked condition) and the unfamiliar-prefer group (infants showing mother’s face preference lower than 50%), the mean number of unmasked people with statical significance was reported.
