# Supplementary material for: Variations in face experiences during the COVID-19 pandemic affect infants’ preference for their mother’s face
Source: PLoS One. 2026 Feb 11;21(2):e0340620. doi: 10.1371/journal.pone.0340620 (PMC12893562; doi:10.1371/journal.pone.0340620)
Supplement: Attachment — Submitted filename: PONE-D-24-49778.pdf [file pone.0340620.s004.pdf]

Peer Review of the Manuscript
“Alteration of Face Experiences During the COVID-19 Pandemic Affects Infants' Preference for the Mother's Face”

Dear authors,

Thank you for the opportunity to peer review this interesting article. The study investigates how altered social environments caused by the COVID-19 pandemic influence infants’ face processing. Specifically, it examines whether 5- to 8-month-old infants born in urban (Tokyo) versus rural (provincial cities in Japan) areas demonstrate differences in visual preference for their mother’s face under masked and unmasked conditions. The results suggest that facial experience, shaped by varying degrees of exposure to unmasked individuals, modulates children’s preferences. In Tokyo, infants show a preference for the mother’s face in a masked condition, whereas in the provincial areas they prefer the mother’s face over that of a stranger, regardless of whether the faces were wearing a mask or not. Furthermore, infants in the provincial areas prefer their mother without a mask compared to children in Tokyo.

General comments: The article explores an important topic for understanding the development of children born during the COVID-19 pandemic. The study is relevant and well-designed. However, it could benefit from a deeper integration of the findings into broader developmental theories and a more in-depth discussion of potential confounding factors. The findings are interesting but sometimes over-generalized, especially given the small sample size (Experiment 1,2,3) and retrospective nature of the questionnaire data (Experiment 3). Overall, the research provides valuable insights and raises important questions about the impact of pandemic-related social changes.

Major points

Introduction

Research Question: While the study compares infants from Tokyo and provincial areas, the RQ itself focus primarily on the general effects of face mask exposure on infants’ preferences for their mothers’ faces without specifically addressing how regional factors (e.g., the extent of mask use or social environments) might influence these preferences. To better align the RQ with the study’s analysis, I would recommend rephrasing it to include a direct reference to potential regional differences in the context of the pandemic and face mask exposure. This would make exploring these factors more explicit and linked to the central investigation of the study.

Hypotheses: The hypothesis is generally clear regarding its logic and predictions. It sets up a straightforward expectation: Infants will prefer their mother’s face, whether masked or unmasked. What could be clarified:

- The second hypothesis (p.4, l. 98–100), suggesting that infants would prefer their mothers’ faces with masks, may need clarification. Since the study focuses on household data, where mothers are less likely to wear masks, it’s unclear whether this hypothesis accurately reflects the infants’ exposure. To strengthen it, consider clarifying the extent of masked face

exposure within the household or reframing the hypothesis to better align with the study's context.

- The study collects data from an urban and a provincial area. However, in the introduction, the hypothesis does not clearly explain how these two different data collections could influence the results. A brief explanation of the hypothesis concerning provincial versus urban data is given later (p.10, l. 237-241), but this could be confusing for the reader.

Preregistration: To further improve the study, it would be helpful to address whether the study was preregistered.

Methods

Participants:

- Discuss potential biases in participant recruitment, such as socioeconomic factors, which might influence the results (if the sample is from a high SES, the use of face mask might be different than from a sample with a low SES).
- The sample sizes are relatively small, especially for Experiment 3. Although justified by the pandemic’s constraints, acknowledging this limitation and its impact on generalizability in the discussion might be useful. You should include a power analysis to strengthen the base of the study.
- The article does not mention whether the participants were paid/rewarded to take part in the study.

Controls for bias:

- While efforts were made to standardize the stimuli, differences in facial features might influence infants’ preferences. The selection of images (mothers’ and strangers’ faces) is described (p.6, l. 140-142) but it is unclear if facial similarity between the stranger and mother was systematically controlled to avoid confounding.
- A white mask and a brown notebook (p. 6, l. 135) were chosen for the experiment; however, there is no mention of possible color biases.

Clarity of experimental design:

- The inclusion of a control condition (face covered with notebook) is a valid choice. However, the reason for excluding this choice from Experiment 2 is not clear. Between lines 241-244 (p.10) it is stated that this was excluded because the preference for mothers’ faces was found only when they wore masks. However, it would have been interesting to explore the object-occluded condition for Experiment 2 as well, especially given the small sample of Experiment 1.
- For Experiment 1, each infant was shown each of the three conditions for two 15-second trials for a total of 90 seconds (p.7, l. 164-165). For Experiment 2, each infant was shown the two conditions for two 15-second trials for a total of 60 seconds (p.12, l. 277-279). The longer duration of the trials in Experiment 1 could give the children more time to process the images, potentially influencing their ability to show a preference.
- The questionnaire (Experiment 3) has many questions relevant to the study. However, no demographic data was collected about the family nor about who filled in the questionnaire. Moreover, information on caregiver occupations and who the infant spends the most time with could clarify how face exposure influences preferences. For example, infants spending

more time with mothers may develop stronger preferences for their faces, while those spending time with other caregivers might not. For international readers, it may not be clear how maternity/paternity leave works in Japan.

- The measure of "exposure to unmasked faces" focuses exclusively on home environments, excluding potential interactions outdoors or in public spaces. For the readers, it may not be clear whether there was outdoor exposure. Did infants in that pandemic period never interact in outdoor places (e.g., doctor, shops, parks)? If infants have been mainly at home, where mothers do not wear a mask, how should this affect the infants' behaviour? If, on the other hand, there was minimal outdoor interaction, could mask use differ between urban and provincial areas in public spaces? Furthermore, the "exposure to unmasked faces" measure does not consider the duration or quality of interactions, could this affect the face preference?
- Do provincial and city mothers differ? Could the observed differences between urban and provincial infants' face preferences be influenced by variations in maternal interaction time? For example, urban mothers may work outside the home more frequently, leading to less exposure to their unmasked faces compared to provincial mothers. Have you considered this as a potential confounding factor, and could it be addressed in the discussion or future research?

Data Analysis: The data analysis is appropriate for the research questions. However, it might be useful to explain why the Bonferroni correction (p.8, l. 187) was chosen over other methods.

General Discussion and Results

The general discussion section is well structured and covers the main points in relation to the research question. However, it is long and sometimes repetitive. Some transitions between paragraphs could be smoother to improve the overall flow of the argument, for example:

- *"…infants in Tokyo showed a significant preference for their mother's faces … with face masks" (*p. 16, l.393) and "*We found that the infants in Tokyo and other provincial cities showed a significant preference for their mother's faces when presented with images of faces wearing masks"* (p.16, l. 401). These two passages present the same finding, but in two different paragraphs. You could try to condense these sentences into the same paragraph.

Overgeneralization: Some conclusions may overestimate the results, especially in view of the small sample size and the limited information on the individual experiences of the infants. For example:

- Lines 427-429 (p.18) state that infants with limited exposure to unmasked faces may prefer an unfamiliar female face because they already have sufficient familiarity with their mother's face. However, this explanation is an overgeneralization, as mothers in both Tokyo and provincial areas did not wear masks at home. All infants likely had the same exposure to their mother's unmasked face in the home environment. The real factor that could differ is the amount of time spent with their mother, but this data was not collected.

Interpretation of Findings

- The interpretation of the results is accurate, but it would be useful to explore more the limitations of the study. For example, the impact of sample size and demographic factors could be discussed more explicitly in relation to how these may have influenced the results.

- The discussion addresses environmental factors that influence face processing. However, some claims, such as children's adaptation to masked environments, could benefit from further integration of developmental theories or comparisons with other studies in similar contexts (e.g. https://doi.org/10.1348/026151001166047, https://doi.org/10.1177/23727322211068007)

Integration with literature: The discussion integrates the findings with existing literature well. However, some interpretations are speculative, such as the novelty preference hypothesis for unfamiliar faces in the Unmasked condition. Consider providing additional evidence or suggesting future studies to address these speculations.

Minor Comments

Formal mistake: There are some syntax and grammatical mistakes in the article. I suggest a review by a native speaker.

Terms to review

- Please avoid the term "developmental change" (p. 9, l. 201). Since you are analysing cross-sectional data, you cannot make statements about change only about differences between age groups.

Unclear Passages

- The sentence "Additionally, previous studies have demonstrated..." (lines 57–60) could benefit from additional references to support the claim about the influence of social environments on face processing development.
- The passage "Wearing face masks**,** particularly in Japan**,** might have a significant impact on the development of face perception**.** In Japan**,** as well as in other countries**,** wearing face masks was ubiquitous in public areas if not at home**"** (lines 84–85) is a somewhat unclear. "**Particularly in Japan**" suggests that mask-wearing was significant in Japan, but the subsequent "**as well as in other countries**" implies a broader, more universal experience. To clarify this argument:
    - If the focus is on Japan, provide references to support the claim that mask-wearing in Japan was particularly impactful.
    - If the aim is also to make a global comparison, you could state which other countries/areas are being referred to. In this way, the comparison would be clear and in context.
- The passage (p. 18, l 433-437) is unclear, particularly the sentence "*unmasked faces exposed to infants showing a preference for a novel unfamiliar female were predominantly those belonging to their own mothers"*. The idea behind the passage is that, due to the high degree of familiarity with the mother's face, children may not find the mother's face so interesting and, therefore, may show a preference for an unfamiliar face, even if this face wears a mask. Reformulate the passage to make it easier to follow for the readers.
