# Supplementary material for: Variations in face experiences during the COVID-19 pandemic affect infants’ preference for their mother’s face
Source: PLoS One. 2026 Feb 11;21(2):e0340620. doi: 10.1371/journal.pone.0340620 (PMC12893562; doi:10.1371/journal.pone.0340620)
Supplement: Attachment — Submitted filename: responseToReviewers_plosone_r1.docx [file pone.0340620.s006.docx]

Reviewer #1: see Review Document

**Major points**

**Introduction**

Research Question: While the study compares infants from Tokyo and provincial areas, the RQ itself focus primarily on the general effects of face mask exposure on infants’ preferences for their mothers’ faces without specifically addressing how regional factors (e.g., the extent of mask use or social environments) might influence these preferences. To better align the RQ with the study’s analysis, I would recommend rephrasing it to include a direct reference to potential regional differences in the context of the pandemic and face mask exposure. This would make exploring these factors more explicit and linked to the central investigation of the study.

***Response: Thank you for your comment. Accordingly, we have revised the introduction section to clarify how regional differences might influence the results (page 4, line 85–page 5, line 88).***

Hypotheses: The hypothesis is generally clear regarding its logic and predictions. It sets up a straightforward expectation: Infants will prefer their mother’s face, whether masked or unmasked. What could be clarified:

- The second hypothesis (p.4, l. 98–100), suggesting that infants would prefer their mothers’ faces with masks, may need clarification. Since the study focuses on household data, where mothers are less likely to wear masks, it’s unclear whether this hypothesis accurately reflects the infants’ exposure. To strengthen it, consider clarifying the extent of masked face exposure within the household or reframing the hypothesis to better align with the study's context.
- The study collects data from an urban and a provincial area. However, in the introduction, the hypothesis does not clearly explain how these two different data collections could influence the results. A brief explanation of the hypothesis concerning provincial versus urban data is given later (p.10, l. 237-241), but this could be confusing for the reader.

***Response: Thank you for your comments. Accordingly, we have revised the introduction section to clarify the hypotheses for the masked condition (page 6, lines 111–117) and how differences in regions might influence the results (page 4, line 85– page 5, line 106).***

Preregistration: To further improve the study, it would be helpful to address whether the study was preregistered.

***Response: We have added a sentence mentioning that this study was not preregistered. (page 32, line 712)***

**Methods**

Participants:

- Discuss potential biases in participant recruitment, such as socioeconomic factors, which might influence the results (if the sample is from a high SES, the use of face mask might be different than from a sample with a low SES).
- The sample sizes are relatively small, especially for Experiment 3. Although justified by the pandemic’s constraints, acknowledging this limitation and its impact on generalizability in the discussion might be useful. You should include a power analysis to strengthen the base of the study.
- The article does not mention whether the participants were paid/rewarded to take part in the study.

***Response: In our study, only infants whose parents were interested in participating and who contacted the laboratory were included. Therefore, participants were likely to have a middle-class status. However, considering that Japan had persistently high rates of reported face mask use in public areas (> 90%; OECD, 2021), we believe it is unlikely that socioeconomic factors influenced the results. We have added sentences mentioning this point to the Participants section (page 8, lines 162-166). We also added sentences regarding this as limitations and future directions in the Discussion section (page 24, lines 550–553).***

***Accordingly, we have added the results of a power analysis for the behavioral experiments (page 7, line 148– page 8, line 159). The results of the power analysis revealed that the number of participants was sufficient to conduct the statistical analysis. However, we have added sentences regarding the relatively small sample size as a limitation (page 24, line 549).***

***We added some sentences to the Participants section mentioning that the participants were rewarded for their involvement in the study. (page 8, lines 166–167).***

Controls for bias:

- While efforts were made to standardize the stimuli, differences in facial features might influence infants’ preferences. The selection of images (mothers’ and strangers’ faces) is described (p.6, l. 140-142) but it is unclear if facial similarity between the stranger and mother was systematically controlled to avoid confounding.
- A white mask and a brown notebook (p. 6, l. 135) were chosen for the experiment; however, there is no mention of possible color biases.

***Response: We have added some sentences to refer to controlling for similarity between the mother’s face and a stranger’s face. (page 8, line 181– page 9, line 183)***

***Traditionally, previous studies have investigated infants’ preferences for primary color stimuli (e.g., red, green, yellow, and blue) but not for achromatic stimuli. Only one study (Franklin et al., 2008) examined infants’ visual preference for colors (red, yellow, green, blue, pink, purple, orange, and brown) by presenting two colors simultaneously. They revealed that infants showed a significantly shorter looking time for brown compared to the mean total looking time, suggesting no preference and/or bias for brown. Therefore, we believe that color preference had little to no influence on our results. (page 8, lines 176–-177).***

Clarity of experimental design:

- The inclusion of a control condition (face covered with notebook) is a valid choice. However, the reason for excluding this choice from Experiment 2 is not clear. Between lines 241-244 (p.10) it is stated that this was excluded because the preference for mothers’ faces was found only when they wore masks. However, it would have been interesting to explore the object-occluded condition for Experiment 2 as well, especially given the small sample of Experiment 1.

***Response: The main focus of this study was to examine whether mask-wearing influenced infants’ visual preference for their mother’s face over that of an unfamiliar female during the COVID-19 pandemic. We addressed this by comparing the preference scores between the unmasked and masked conditions.***

***During the pandemic, mothers’ faces were commonly masked and unmasked in everyday life in Tokyo and provincial regions in Japan, and thus considered familiar. We hypothesized that infants born and raised during the COVID-19 pandemic would show a preference for their mother’s face regardless of whether she was wearing a mask. The notebook-occluded condition was introduced in Experiment 1 as a control to present a clearly unfamiliar facial stimulus. Unlike a masked face, which is socially relevant, a notebook-occluded face would not resemble the typical visual input infants experience. This was expected to be equally unfamiliar to infants in both regions. Considering that infants in Tokyo showed no preference for their mother’s face when it was occluded by a notebook in Experiment 1, we reasoned that infants in provincial cities would also not show a preference in this condition. Instead, we focused on directly comparing the masked and unmasked conditions, which were central to our hypothesis. We acknowledge that including the occluded condition in both experiments may have strengthened our interpretation, and we appreciate the reviewer’s suggestion. We added sentences to clarify this point. (page 13, lines 299–306)***

- For Experiment 1, each infant was shown each of the three conditions for two 15-second trials for a total of 90 seconds (p.7, l. 164-165). For Experiment 2, each infant was shown the two conditions for two 15-second trials for a total of 60 seconds (p.12, l. 277-279). The longer duration of the trials in Experiment 1 could give the children more time to process the images, potentially influencing their ability to show a preference.

***Response: If the longer total duration in Experiment 1 than in Experiment 2 affected the infants’ visual preference, we could predict that infants in Tokyo would show a visual preference for their mother’s face in all three conditions, as they would have more time to process a pair of faces (mother and stranger). However, we found that infants born and raised in Tokyo did not show a preference for their mother’s face in the unmasked and object-occluded conditions. Therefore, we believe that no potential influence would be observed.***

- The questionnaire (Experiment 3) has many questions relevant to the study. However, no demographic data was collected about the family nor about who filled in the questionnaire. Moreover, information on caregiver occupations and who the infant spends the most time with could clarify how face exposure influences preferences. For example, infants spending more time with mothers may develop stronger preferences for their faces, while those spending time with other caregivers might not. For international readers, it may not be clear how maternity/paternity leave works in Japan.

***Response: In Japan, most working mothers (approximately 80–85%) take parental leave, with approximately 90% of them taking leave for longer than 6 months. In contrast, only approximately 10% of fathers take parental leave, although this number has been gradually increasing. Furthermore, the duration of leave taken by fathers tends to be significantly shorter than that taken by mothers, with approximately 90% of fathers taking less than 3 months of leave. Consequently, infants under 1 year of age in Japan spend most of their early lives with their mothers, regardless of whether their mothers are employed. Therefore, that most questionnaire respondents in Experiment 3 were mothers can be reasonably assumed.***

***We added sentences that explain how parental leave works in Japan. (page 19, lines 432–441)***

- The measure of “exposure to unmasked faces” focuses exclusively on home environments, excluding potential interactions outdoors or in public spaces. For the readers, it may not be clear whether there was outdoor exposure. Did infants in that pandemic period never interact in outdoor places (e.g., doctor, shops, parks)? If infants have been mainly at home, where mothers do not wear a mask, how should this affect the infants’ behaviour? If, on the other hand, there was minimal outdoor interaction, could mask use differ between urban and provincial areas in public spaces? Furthermore, the “exposure to unmasked faces” measure does not consider the duration or quality of interactions, could this affect the face preference?

***Response: For most of the period in which Experiment 1 was conducted (June–November 2011), a state or quasi-state of emergency was declared in Tokyo. In contrast, during most of the period when Experiment 2 was conducted (January–March 2022 and December–February 2022), the provincial regions were not under such declarations. Considering these situational differences, the families of the infant participants in Tokyo were potentially more likely to refrain from going out than those in the provincial regions. Therefore, the possibility that the number of masked faces that infants saw in public differed between urban and provincial regions cannot be ruled out completely.***

***However, during both experimental periods, although the Japanese or local governments requested that people limit their outings, no strict lockdowns such as seen in other countries were imposed. Thus, Japanese people, including families with infants, were still able to go out for essential activities (e.g., shopping, medical visits, or going to parks), and during these outings, people generally wore face masks. Therefore, it can be reasonably assumed that infants were exposed to a large number of masked faces once outside the home and that this experience was broadly similar across Tokyo and the provincial regions. We added sentences referring to this point. (page 18, lines 409–421).***

***However, we did not directly collect data on the infants’ social interactions outside their homes. Similarly, we did not measure the duration and quality of interaction because of limitations inherent in the retrospective design of Experiment 3 (i.e., it would have been difficult for parents to recall those details accurately).***

***While we acknowledge that the duration and quality of interaction could, in theory, influence face preferences, currently available evidence suggests that such effects are limited or context-dependent. For example, Juvrud et al. (2019) found no clear effect of caregiving arrangements (female-only versus distributed caregivers) on infants’ face gender preferences. Nonetheless, future studies that directly quantify these interactions may provide further insights. (page 24, lines 554–558)***

- Do provincial and city mothers differ? Could the observed differences between urban and provincial infants’ face preferences be influenced by variations in maternal interaction time? For example, urban mothers may work outside the home more frequently, leading to less exposure to their unmasked faces compared to provincial mothers. Have you considered this as a potential confounding factor, and could it be addressed in the discussion or future research?

***Response: As referred to a response to the reviewer’s comment, in this study, variation in the amount and/or quality of maternal interaction without face masks may have been relatively minimal, as mothers are typically the primary caregivers in most Japanese families, regardless of the region. However, as the reviewer noted, the generalizability of the present findings to populations or regions in which caregiving roles show greater variation should be interpreted with caution. We have added sentences referring to this point as a limitation. (page 24, lines 554–558)***

Data Analysis: The data analysis is appropriate for the research questions. However, it might be useful to explain why the Bonferroni correction (p.8, l. 187) was chosen over other methods.

***Response: We applied the Bonferroni correction in this study rather than alternative methods (e.g., Holm and False Discovery Rate) for the following reasons. First, as noted by the reviewers, sample sizes in infant perception research tend to be small owing to practical constraints, although the current sample sizes were determined based on power analyses. Although small samples increase the risk of spurious findings because of random variation, the highly conservative Bonferroni correction helps reduce the likelihood of Type I error (false positives). Second, the current study involved a small predefined set of comparisons (three for Experiment 1 and two for Experiment 2). Under such conditions, the increase in Type II errors (false negatives) associated with the Bonferroni correction is minimal compared to that of other correction methods. Moreover, the Bonferroni correction is widely used in developmental research on infant perception, making it a well-established and transparent choice for controlling family-wise errors.***

***We added sentences explaining these to the Data analysis section. (page 10, line 230–page 11, line 240)***

**General Discussion and Results**

The general discussion section is well structured and covers the main points in relation to the research question. However, it is long and sometimes repetitive. Some transitions between paragraphs could be smoother to improve the overall flow of the argument, for example:

- “…infants in Tokyo showed a significant preference for their mother’s faces … with face masks” (p. 16, l.393) and “We found that the infants in Tokyo and other provincial cities showed a significant preference for their mother’s faces when presented with images of faces wearing masks” (p.16, l. 401). These two passages present the same finding, but in two different paragraphs. You could try to condense these sentences into the same paragraph.

***Response: Thank you very much for your suggestion. In response to the reviewer’s comment, we have revised the General Discussion section to eliminate redundancy.***

Overgeneralization: Some conclusions may overestimate the results, especially in view of the small sample size and the limited information on the individual experiences of the infants. For example:

- Lines 427-429 (p.18) state that infants with limited exposure to unmasked faces may prefer an unfamiliar female face because they already have sufficient familiarity with their mother’s face. However, this explanation is an overgeneralization, as mothers in both Tokyo and provincial areas did not wear masks at home. All infants likely had the same exposure to their mother’s unmasked face in the home environment. The real factor that could differ is the amount of time spent with their mother, but this data was not collected.

***Response: As the reviewer noted, we did not collect specific information on the exact amount of time mothers spent with their infants without face masks. It was also difficult to determine this amount of time through a questionnaire. We have added sentences referring to this point and mentioned some future directions. (page 23, lines 523–527)***

Interpretation of Findings

- The interpretation of the results is accurate, but it would be useful to explore more the limitations of the study. For example, the impact of sample size and demographic factors could be discussed more explicitly in relation to how these may have influenced the results.

***Response: Thank you very much for your suggestion. In response to the reviewer’s comment, we have added the limitations of this study to the General Discussion. (page 24, lines 540–561)***

- The discussion addresses environmental factors that influence face processing. However, some claims, such as children’s adaptation to masked environments, could benefit from further integration of developmental theories or comparisons with other studies in similar contexts (e.g. https://doi.org/10.1348/026151001166047, https://doi.org/10.1177/23727322211068007)

***Response- Thank you for your suggestion. Accordingly, we have referred to some previous studies in the Discussion section. (page 22, lines 501–504)***

Integration with literature: The discussion integrates the findings with existing literature well. However, some interpretations are speculative, such as the novelty preference hypothesis for unfamiliar faces in the Unmasked condition. Consider providing additional evidence or suggesting future studies to address these speculations.

***Response- Thank you for your comment. Based on your suggestion, we have added sentences mentioning the potential for future research. (page 23, lines 523–527)***

**Minor Comments**

Formal mistake: There are some syntax and grammatical mistakes in the article. I suggest a review by a native speaker.

***Response- Our manuscript has undergone English proofreading.***

Terms to review

- Please avoid the term “developmental change” (p. 9, l. 201). Since you are analysing cross-sectional data, you cannot make statements about change only about differences between age groups.

***Response- We replaced the term “developmental change” with “developmental trend.” (page 11, line 256)***

Unclear Passages

- The sentence “Additionally, previous studies have demonstrated...” (lines 57–60) could benefit from additional references to support the claim about the influence of social environments on face processing development.

***Response- We have added references to the end of this sentence. (page 3, line 53)***

- The passage “Wearing face masks, particularly in Japan, might have a significant impact on the development of face perception. In Japan, as well as in other countries, wearing face masks was ubiquitous in public areas if not at home” (lines 84–85) is a somewhat unclear. “Particularly in Japan” suggests that mask-wearing was significant in Japan, but the subsequent “as well as in other countries” implies a broader, more universal experience. To clarify this argument:
- If the focus is on Japan, provide references to support the claim that mask-wearing in Japan was particularly impactful.
- If the aim is also to make a global comparison, you could state which other countries/areas are being referred to. In this way, the comparison would be clear and in context.

***Response- Thank you for your suggestion. We have revised the relevant paragraph to clarify our argument. (page 4, lines 75–77)***

•

- The passage (p. 18, l 433-437) is unclear, particularly the sentence “unmasked faces exposed to infants showing a preference for a novel unfamiliar female were predominantly those belonging to their own mothers”. The idea behind the passage is that, due to the high degree of familiarity with the mother’s face, children may not find the mother’s face so interesting and, therefore, may show a preference for an unfamiliar face, even if this face wears a mask. Reformulate the passage to make it easier to follow for the readers.

***Response- According to your comment, we have revised the sentences. (page 23, lines 521–523)***

Reviewer #2: The authors present very interesting findings based on two experimental studies and a follow-up survey on infant’s preferences for mother’s face during COVID-19 pandemic. Below are my suggestions for further improvement of the manuscript before I can endorse publication:

1. You are referring to a variety of facial experiences without necessarily implying a switch or alternation. Perhaps, a word like "variation" might better convey your intended meaning. Please consider changing the beginning of the title as “variation in face experiences ……”

***Response: Thank you very much for your suggestion. Accordingly, we have revised the title of this study.***

2. Please elaborate on how the sample size was determined for Experiments 1 and 2. Did you perform a power analysis?

***Response: We added some sentences to elaborate on the sample size estimation in Experiments 1 and 2 (page 7, line 148– page 8, line 159).***

3. Lines 218- 225: Please elaborate further on how previous studies differed from the present study, and how these differences might explain the inconsistencies between your findings and those of earlier reports.

***Response: We have added sentences to elaborate on the differences between the present and previous studies and the possibility of explaining the inconsistent results. (page 12, lines 274–279)***

4. Lines 164 and 277: Did I understand correctly that the experiments consisted of only 2 trials? Could you please include the rationale for having very few trials?

***Response: In the preferential looking procedure, a method is used to assess infants’ visual perception based on their spontaneous preference for certain characteristics of visual stimuli, in which two visual stimuli (one target and one non-target) are presented simultaneously. Typically, two trials are conducted with the positions of the stimuli (left or right) switched between trials to control for potential side bias (i.e., infants may look longer at one side of the screen for reasons unrelated to the stimuli themselves). Theoretically, more than two trials could be conducted for a given stimulus pair. However, this is often not feasible for the following reasons. First, infant studies are subject to specific constraints, including infants’ short attention spans and their susceptibility to fatigue or fussiness. Second, increasing the number of trials can lead to habituation to the stimulus, which may diminish infants’ responsiveness and affect the results. Considering these limitations, we determined that a two-trial design was the most appropriate. Similar two-trial designs have been commonly used in previous infant studies that employed a preferential-looking paradigm.***

5. Line 176- 181: It is great that you have ensured interobserver reliability, however, why is it that only 40% of the data was analysed by the second observer, rather than two independent observers analyzing the entire dataset? Ideally, the second observer should have completed the analysis of the remaining 60%. If this is not feasible, the rationale behind this decision must be addressed and referred to as a limitation in the discussion section, as this can potentially influence the results.

***Response: Thank you for your comment. We calculated interobserver reliability based on 40% of the data. This decision was made primarily because of limited research personnel and time, as the analysis was conducted manually and required considerable effort and training for each coder. Although we agree that having two observers analyze the entire dataset would be ideal, it was not feasible under the current constraints. We acknowledge this as a limitation in the Discussion section of the revised manuscript.***

***Nevertheless, calculating interobserver reliability on a subset of the data is a widely accepted practice in infant visual preference research. For example, prior studies have typically reported interobserver reliability for 20–30% of the data (e.g., Yamashita et al., 2011; Otsuka et al., 2008; Quinn et al., 2008; Safar et al., 2020 at around 20%; Dupierrix et al., 2014; Bulf et al., 2015 at approximately 30%). In some cases, even smaller subsets have been used. For example, Sakuta et al. (2018) reported interobserver agreement on only 10% of their data, yet achieved a high correlation (r = .87). Furthermore, studies that analyzed the full dataset have reported similar correlation values (e.g., r ≈ .90; Yang et al., 2015; Damon et al., 2015; Kelly et al., 2005), suggesting that analyzing a smaller subset does not necessarily compromise reliability.***

***We have added a statement in the Discussion acknowledging the partial double-coding as a limitation that may potentially affect the generalizability of the interobserver reliability estimates. (page 24, lines 559–561)***

6. Lines 193: 196: In this section, there are some issues related to interpretation of the non-significant findings. P-values should not be interpreted to confirm the null, as these are likely inconclusive findings (considering the power& sample size). I suggest either following up these findings with Bayesian statistics or rephrasing the sentences in a way that they do not confirm the null.

***Response: Thank you for your important comment. We have revised the sentences to mention the non-significant results in Experiment 1 (page 11, lines 245–248).***

Minor:

Line 97: Don’t start the sentence with also

***Response- We have revised the sentence.***

Lines 104- 107: Please rephrase this sentence for an improved grammar and clarity.

***Response- We have revised the sentence. And the manuscript has undergone proofreading. (page 6, lines 123–126)***

Line 153: “brown-colored notebook”, which appears green in the image in Figure 1.

***Response- Thank you for pointing that out. We confirmed that in the original image of Figure 1, the color of the notebook was brown. It is possible that the color changed during the conversion process to PDF.***

Line 199: Please include cohen’s d here too.

***Response- We have added the value of Cohen’s d to the Result Section. (page 11, lines 250–252)***

Line 239: Replace the word “facts” with “findings”

***Response- We have replaced the word “facts” with “findings.” (page 13, line 296)***
