# Supplementary material for: Variations in face experiences during the COVID-19 pandemic affect infants’ preference for their mother’s face
Source: PLoS One. 2026 Feb 11;21(2):e0340620. doi: 10.1371/journal.pone.0340620 (PMC12893562; doi:10.1371/journal.pone.0340620)
Supplement: Attachment — Submitted filename: responseToReviewers_plosone_r2_251116.docx [file pone.0340620.s007.docx]

Reviewer #1: Thank you very much for sharing the revised version of your manuscript. We can see that you have implemented all of our previous comments, which has improved the overall clarity of the paper. We still have a few remaining points that we would like to raise:

1) Hypothesis (126–131, page 6):

The description of the findings by Miyazaki and Kawahara [24] and the subsequent hypothesis are somewhat confusing. The cited study reports that observers rated faces as less attractive when occluded by a mask, but not when occluded by a control object (e.g., a notebook). Based on this finding, one would expect that infants might prefer their mother’s face occluded by an object rather than by a mask, which seems the opposite of what is stated in your hypothesis. As currently written, it is not entirely clear how your hypothesis logically follows from the cited finding. Perhaps you could clarify this link or adjust the phrasing to better reflect the underlying rationale.

***Response- Thank you for your insightful comment. In our manuscript, we cited Miyazaki and Kawahara (2016) to demonstrate that occlusion by face masks, rather than by other objects, has specific effects on face perception, rather than to emphasize the influence on facial attractiveness. However, as the reviewer pointed out, the previous wording could be interpreted as implying that changes in perceived facial attractiveness caused by face masks might influence infants’ preferences for their mothers’ faces. To avoid this misunderstanding, we have revised the paragraph to clarify that the citation of Miyazaki and Kawahara (2016) was intended to highlight the mask-specific nature of face perception. (page 6, lines 126-131)***

2) Power analysis:

You mention that the power analyses were conducted for t-tests, but the largest statistical model used in the study was an ANOVA. Could you please clarify whether the power was sufficient for this more complex model as well?

***Response- Thank you for your helpful comment. To confirm whether the statistical power was sufficient for the more complex model (one-way ANOVA), we additionally conducted a post hoc power analysis using G*Power 3.1 (Faul et al., 2007). This analysis revealed that the achieved power was 1.00 (α = .05, f = 0.50, η_p_^2^ = 0.201, N = 22), indicating adequate sensitivity to detect a large effect. We have added some sentences to clarify this point. (page 8, lines 162-166)***

3) SES argument (333–334, page 15):

You wrote, “All participants came from families with a middle-class socioeconomic background in Japan.” However, as you mentioned on page 8, this is only an assumption based on the voluntary nature of participation. It might be important to make this explicit. The same applies to lines 551–553: “This is because they could be related to mask-wearing behavior, although the participants of this study had a middle-class socioeconomic background.”

***Response- Thank you for your helpful comment. We agree that the statement should explicitly state that participants’ socioeconomic background was an assumption based on the voluntary nature of participation. Accordingly, we have revised the sentence to clarify this point. (page 15, lines 343-345; page 24, lines 564-565)***

4) SES argument (162-167, page 8):

You discuss socioeconomic status (SES) in relation to mask frequency, but SES could also lead to differences in face perception itself. In other words, the same frequency of exposure to masked faces might have different effects on children from different SES backgrounds. It would be interesting if you could briefly discuss this possibility.

***Response- Thank you very much for this insightful comment. To the best of our knowledge, a previous study reported no effect of rearing background, including socioeconomic status (SES), on infants’ visual attention to faces (Pyykkö et al., 2019). Therefore, although we did not directly assess SES in the present study, we believe that it did not affect infants’ preferences for face perception. We have described this point in the Participants section (page 8, lines 171-173) and the Discussion section (page 24, lines 562-567) in the revised manuscript.***

5) Typo (409, page 18): There seems to be a small typo, I assume the year should be 2021 and not 2011.

***Response- Thank you very much for this comment. We have revised the typo concerning the year. (page 18, line 421)***
